# Self-assembly of iron oxide precursor micelles driven by magnetic stirring time in sol–gel coatings†

**DOI:** 10.1039/c9ra03283e

**Published:** 2019-06-04

**Authors:** J. López-Sánchez, A. Serrano, A. del Campo, M. Abuín, E. Salas-Colera, A. Muñoz-Noval, G. R. Castro, J. de la Figuera, J. F. Marco, P. Marín, N. Carmona, O. Rodríguez de la Fuente

**Affiliations:** Instituto de Magnetismo Aplicado, UCM-CSIC-ADIF 28230 Madrid Spain jesus.lopez@ucm.es; Instituto de Cerámica y Vidrio, ICV-CSIC 28049 Madrid Spain; SpLine, Spanish CRG BM25 Beamline, ESRF-The European Synchrotron 38000 Grenoble France; Instituto de Ciencia de Materiales de Madrid, ICMM-CSIC 28049 Madrid Spain; Instituto de Sistemas Optoelectrónicos y Microtecnología, ISOM-UPM 28040 Madrid Spain; Instituto de Química Física “Rocasolano”, IQFR-CSIC 28006 Madrid Spain; Departamento de Física de Materiales, Universidad Complutense de Madrid (UCM) 28040 Madrid Spain

## Abstract

The purpose of this work is to fabricate self-assembled microstructures by the sol–gel method and study the morphological, structural and compositional dependence of ε-Fe_2_O_3_ nanoparticles embedded in silica when glycerol (GLY) and cetyl-trimethylammonium bromide (CTAB) are added as steric agents simultaneously. The combined action of a polyalcohol and a surfactant significantly modifies the morphology of the sample giving rise to a different microstructure in each of the studied cases (1, 3 and 7 days of magnetic stirring time). This is due to the fact that the addition of these two compounds leads to a considerable increase in gelation time as GLY can interact with the alkoxide group on the surface of the iron oxide precursor micelle and/or be incorporated into the hydrophilic chains of CTAB. This last effect causes the iron oxide precursor micelles to be interconnected forming aggregates whose size and structure depend on the magnetic stirring time of the sol–gel synthetic route. In this paper, crystalline structure, composition, purity and morphology of the sol–gel coatings densified at 960 °C are examined. Emphasis is placed on the nominal percentage of the different iron oxides found in the samples and on the morphological and structural differences. This work implies the possibility of patterning ε-Fe_2_O_3_ nanoparticles in coatings and controlling their purity by an easy one-pot sol–gel method.

## Abstract 2

Sol–gel synthesis of self-assembled iron oxide precursor micelles.

## Graphical abstract

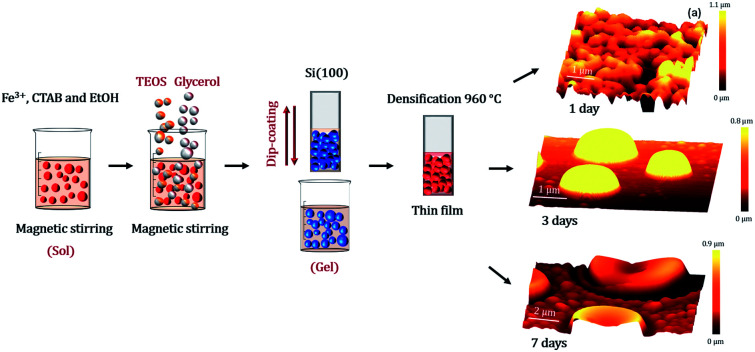

## Introduction

Epsilon iron oxide is a collinear ferrimagnetic material with a Curie transition at around 500 K and a coercive field of 2 T at room temperature.^1–4^ Therefore, it is considered an attractive magnetically hard material which could be used as a composite to produce a new family of oxide-based permanent magnets. Besides, due to its coercivity, the ferromagnetic resonance frequency falls in the milli terahertz range. Several studies have demonstrated the modulation of the resonance frequency depending on different elements substituted in the crystalline structure of the ε-Fe_2_O_3_ phase.^5–8^ Specially in thin film form, this polymorph shows a ferromagnetic and ferroelectric response. Thus it opens the possibility of using it as a room temperature multiferroic in a real device.^9^ Furthermore, this material is the only iron oxide with intrinsic p-type semiconductor behaviour, with a bandgap in the visible range (∼1.9 eV).^10^ Thus, it is a good candidate for catalytic applications such as photo-anode water molecule decomposition in an alcoholic solution.^11^ In addition, there are investigations where this material is also used as a selective NO_2_ sensor in the form of nanopillars.^12^

For the fabrication of this iron oxide phase several methods have been developed to synthesize this material with a high crystallinity: *e.g.* pulsed laser deposition (PLD),^9,13,14^ chemical vapour deposition (CVD)^15^ or sol–gel synthesis.^16–27^ Of these techniques, the sol–gel method is the most widely used to synthesize it. However, the oxide is difficult to obtain, and complications arise when it is microstructured to enhance and/or tune some of the above mentioned properties.

In this regard, we have designed in this work an easy one-pot sol–gel synthesis where different microstructures of ε-Fe_2_O_3_ nanoparticles can be obtained, depending on the magnetic stirring time of the solution. Sol–gel synthesis also stands out among other growth techniques due to the possibility of modifying the micro- and nanostructure according to experimental conditions, homogeneity and particle size of the oxide.^28^ Here, we are able to modify the size and morphology of microstructures fabricated as coatings on Si(100) substrates thanks to the influence of a polyalcohol, glycerol (GLY), and a surfactant, cetyl-trimethylammonium bromide (CTAB). The combined addition of these precursors into the solution provides high stability for the iron oxide nanoparticles. Generally speaking, each synthesis by the sol–gel method needs a specific stirring time by which the hydrolysis and polycondensation processes happen whereas homogeneous solution and sols aggregation are accomplished. Virtually, all previous works that synthesizes ε-Fe_2_O_3_ by the sol–gel method employs CTAB as a surfactant agent for iron oxide precursor micelles.^1,2,29^ To the best of our knowledge, only two studies use GLY^27^ and sucrose^22^ as different type of organic ligand in which it is not intended to modify its crystalline structure or morphology for micropatterning purposes. For this reason, the incorporation of organic ligand as a parameter to take into account in the final structure of sample represents a major breakthrough in the synthesis process.

The aim of this work is to study the structural and morphological evolution of the micelles and how they interconnect among themselves depending on the magnetic agitation time: 1 day, 3 days and 7 days. With the interconnection, these micelles are linked by organic groups, but once formed they do not interact with each other to form large particles. This is achieved by adding the GLY after surrounding the micelles with the CTAB. As the stirring time progresses, the microstructure of the sample is modified, forming large micellar networks. Besides, the compositional ratio of epsilon iron oxide is modified. Our work suggests a new strategy to fabricate different structures by self-assembled iron nanoparticles to obtain, for instance, an enhancement of the catalytic properties due to the increase of the specific surface available to react.

## Preparation and synthesis of samples

**Fig. 1:**
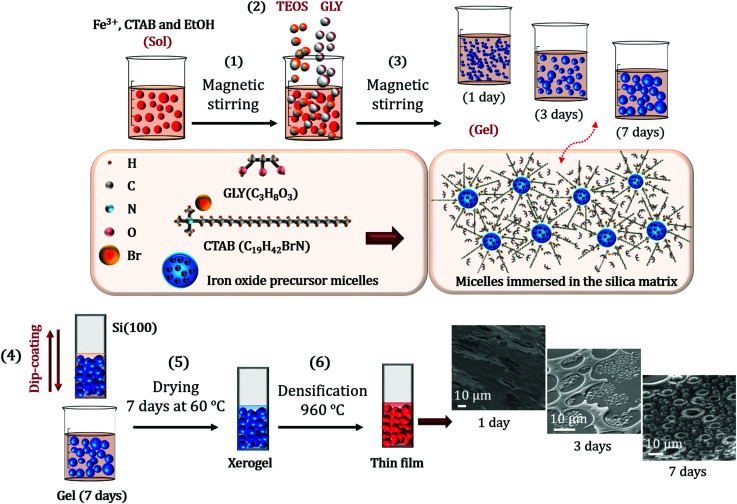
Representation of the synthesis of the self-assembled iron oxide nanoparticles: CTAB surfactant is dissolved in absolute ethanol together with barium nitrate and nona-hydrated iron nitrate. The solution is maintained under vigorous magnetic stirring (1). TEOS is firstly added drop by drop and the hydrolysis and polycondensation processes begin simultaneously to form the silica matrix. After one hour, GLY is incorporated into the solution dropwise (2). The resulting colloidal suspension is kept under magnetic agitation at room temperature for 1, 3, and 7 days (3). Once this time has elapsed, the suspension is deposited on Si(100) substrates by the dip-coating (4). Obtained samples are then placed in an air oven at 60 °C for 7 days to form the xerogel (5). Densification treatments are carried out at 960 °C to obtain the iron oxide nanoparticles (6).

The designed sol–gel synthesis was performed in acid environment with several steps in common with those described in other investigations.^26,27^ Salts of nona-hydrate iron nitrate (Fe(NO_3_)_3_·9H_2_O, Sigma-Aldrich >98%) and barium nitrate (Ba(NO_3_)_2_, Sigma-Aldrich >98%), together with CTAB (C_19_H_42_BrN, Sigma Aldrich >99%) were dissolved in absolute ethanol (CH_3_CH_2_OH, Panreac). The molar concentrations were respectively 1 : 0.002 : 0.1. An illustration of the entire sol–gel synthesis processes is shown in Fig. 1.

The sol was magnetically stirred at high speed for 15 min and the precursor of the silica matrix, tetraethyl orthosilicate (TEOS, SiC_8_H_20_O_4_, Sigma-Aldrich >98%) was subsequently incorporated dropwise with a Fe : Si molar ratio of 1 : 1. The reaction time of the hydrolysis and polycondensation process was supported by 100 μl of nitric acid (HNO_3_, Sigma-Aldrich >70%) afterwards ascertaining that the pH of the solution was close to 1.0. The GLY (C_3_H_8_O_3_, Sigma-Aldrich >99%) was dropwise poured for one hour into the resulting solution with a TEOS : GLY molar ratio of 1 : 1.5, respectively (Fig. 1). Glycerol hydroxyl groups favour the hydrolysis processes and the viscosity of the solution is considerably increased.

The final sol was covered with paraffin film and maintained for several days under magnetic stirring at a vigorous motion. Deposited films on Si(100) (as received) were obtained by dip-coating after 1 day, 3 days and 7 days of stirring time (Fig. 1). The films were then dried in a conventional oven at 60 °C for 7 days and the corresponding xerogels were formed. After this time, densification treatments were carried out at 960 °C under air with a temperature ramp of 1 °C min^−1^ (Fig. 1). The samples obtained consisted of 54 wt% of iron oxide (α- and ε-Fe_2_O_3_) in a SiO_2_ matrix. Synthetic route has been repeated several times obtaining reproducible results.

## Experimental details

Structural and morphological studies of the samples were carried out by X-ray diffraction (XRD), confocal Raman microscopy (CRM) and atomic force microscopy (AFM). The XRD equipment employed was a PANalytical X-ray diffractometer using Cu-Kα (*λ* = 1.5406 Å) radiation. Raman-AFM measurements were carried out using a Witec ALPHA 300RA confocal Raman microscope equipped with a Nd:YAG laser (532 nm). The Raman mappings were performed with a 100× objective lens and a numerical aperture of 0.95 at a 0.7 mW laser power taking spectra every 100 nm with 3 seconds of integration time. AFM mappings were recorded in the same equipment in non-contact mode using an AFM gold-coated silicon probe, NSG30 model provided by NT-MDT (Russia). The tip was about 14–16 microns high, aspect ratio from 3 : 1 to 5 : 1 and curvature radius 10 nm. Cantilever was 125 μm long, 40 μm wide and 4 μm thick, with an elastic constant 40 N m^−1^ and a resonant frequency of 268 kHz. Raman and AFM data were analyzed using the Witec Project Plus software. This system allows studying the same region of the sample for both last characterization techniques. We therefore can know the composition provided by CRM as well as the morphology of the nano- and microstructures obtained by AFM.

Important differences in the morphology and crystal structure were found in some specific samples. Thus, a further structural characterization was performed by Mössbauer spectroscopy and X-ray absorption spectroscopy (XAS), which was needed in order to examine the environment of the different iron cation sites. We first studied these structural properties by integral conversion electron Mössbauer spectroscopy (ICEMS), where data were collected at room temperature in a conventional constant acceleration spectrometer using a ^57^Co(Rh) source and a parallel plate avalanche counter.^30^ All the isomer shift data were referred to the centroid of the spectrum of α-Fe, recorded at room temperature.

XAS experiments were carried out in fluorescence detection mode at the Fe K-edge (7112 eV) in the range 6900–8000 eV at the SpLine BM25A beamline of the ESRF, The European Synchrotron. The samples were placed forming a 45° angle with the incident X-ray direction on a motorized sample positioning setup with four degrees of freedom (*X*, *Y*, *Z* and theta axes). The fluorescence signal was collected using a 13 element Si(Li) solid state detector while iron oxide references XAS signal were collected in transmission mode. The XAS analysis was done on spectra obtained from the average of 5 XAS scans. Data from an iron foil is used as energy calibration of the XAS spectra. XAS data are analysed in accordance with standard procedures using the ATHENA and Artemis program packages.^31^

## Results and discussion

**Fig. 2:**
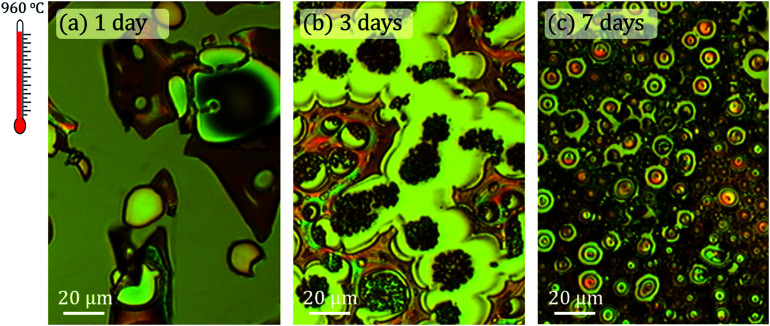
Optical micrographs of the samples synthesized at 960 °C in air for (a) 1 day, (b) 3 days and (c) 7 days of magnetic stirring time. The same scale is maintained in the three cases studied to highlight the abrupt morphological changes that occur as a function of agitation time.

Fig. 2 shows optical micrographs acquired with the CRM system corresponding to samples treated at 960 °C and magnetic agitation times of 1, 3 and 7 days, respectively. Structural and morphological differences are very noticeable. The coating of the 1 day sample is not continuous and consists of different islands tens of microns wide with differences in height between 1–7 μm.

For the 3 days stirring time sample, the irregular morphology disappears and vesicular structures are formed (Fig. 2b). These vesicles are repeated for the whole sample and are located as furrows well delimited by flat tops and irregular surfaces in plane. The arrangement of the vesicles suggests that they detach from these surfaces. Finally, these same ordered microstructures increase in size for the 7 days sample and the flatness of the rest of the surface disappears. In this case, the surface is made up of smaller vesicular structures and it is observed that the sample is relatively rough.

**Fig. 3:**
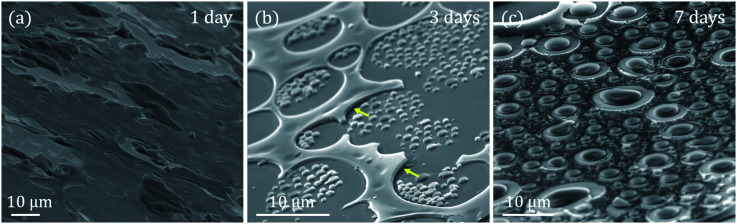
SEM images acquired with an inclination of 60° with respect to the *XY* plane of the samples synthesized at 960 °C for (a) 1 day, (b) 3 days and (c) 7 days of magnetic agitation. Yellow arrows indicate the areas where the substrate coating is detached.

SEM analysis has been carried out to obtain the detailed morphology of these samples (Fig. 3). The SEM images are acquired with an inclination of 60° respect to the electron beam incident to give another perspective of the sample according to the degree of roughness observed. This view shows an irregular coating with no well-defined in-plane structures, finding what seems to be uncovered areas.

The interaction between GLY and CTAB is clear and strongly dependent on the stirring time: the globular-shaped islands increase in size when the stirring time increases (Fig. 2 and 3). Another interesting feature of the 3 days sample is the adherence that these islands present, as they do not seem to be separated from the substrate at any point. This is in contrast with the observation of the rest of the coatings where some raised areas are detected (marked with yellow arrows in Fig. 3b). In this case, there are no substantial differences in height. On the other hand, for the 7 days sample, the islands are larger than the rest of the rough coating compared to the 3 days sample.

**Fig. 4:**
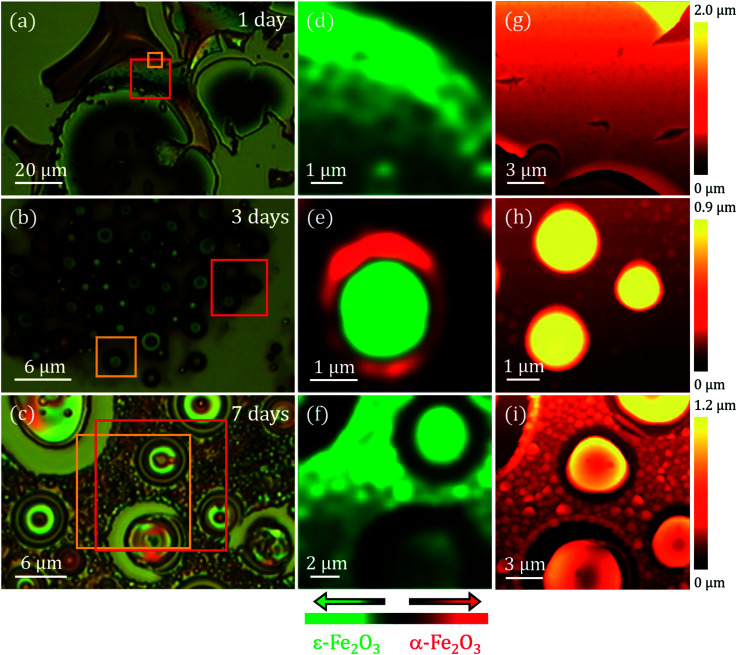
(a–c) Optical micrographs of the coatings treated at 960 °C with magnetic stirring times of 1, 3 and 7 days; (d–f) Raman intensity images in the *XY* plane performed on the regions marked with yellow squares on (a–c), acquiring Raman spectra every 100 nm with an integration time of 3 s. The integration range chosen to obtain the intensity image ranges from 660 cm^−1^ to 750 cm^−1^ for the ε-Fe_2_O_3_ phase (green colour) and from 1310 cm^−1^ to 1340 cm^−1^ for the α-Fe_2_O_3_ phase (red colour); (g–i) AFM topographical images of the surface indicated with a red box on (a–c).

To conclude with the morphological and the surface characterization, AFM measurements were performed in different areas of the samples. The right panel of Fig. 4 illustrates the selected AFM images corresponding to areas indicated with a red square in Fig. 4a–c. For 1 day of stirring, a flat area is shown where we can appreciate how the surface presents a porosity (see details in ESI†). These pores are frequently observed when synthesizing an amorphous silica matrix grown by the sol–gel method.^32^ Typical pore size values reported in other studies are between 5 and 75 nm.^33^ A higher molar concentration of GLY with respect to tetramethyl orthosilicate (TMOS) can generate a higher pore volume, a lower porosity, a higher density and a contraction of the volume of the sample.^33^ These effects appear to be visible in the 1 day sample (see ESI†). In the images corresponding to the 3 and 7 days of stirring samples the porous character of the matrix is not observed and the morphology is completely transformed, with the appearance of islands and particles emerging from the surface of the Si(100) substrate. In the 3 days agitation sample, two types of structure are observed: islands of circular contour with sizes from ∼300 nm to ∼5 μm in width with a height of ∼720 nm and particles that are part of the coating with diameters ranging from ∼190 to ∼540 nm and heights from ∼200 nm to ∼450 nm are clearly distinguished (Fig. 4h). In the case of 7 days of stirring, both the number of islands and their dimensions increase. Island diameters between ∼4 and ∼10 μm have the same height as for 3 days of agitation, but only at the edge of the islands. The upper part of all islands has a depression pointing towards the interior of the island. These depressions extend to ∼300 nm in depth. Also, the most superficial particles increase in size and their diameters range from ∼500 nm to ∼2 μm with heights between ∼200 and ∼500 nm. All these values as well as other roughness parameters are presented in Table 1.

**Table 1:** Calculated parameters from the analysis derived from AFM images. The heights and diameters of the islands of the surface particles corresponding to 3 and 7 days are added

	1 day	3 days	7 days
SA (nm)	44	202	180
SQ (nm)	54	256	229
Island height (nm)	—	720	720
Island diameter (μm)	—	0.3–5	3.3–10
Superficial particle height (nm)	—	200–500	200–500
Superficial particle diameter (nm)	—	190–540	500–1500

The mean height (SA) measures the difference between the height of each point with the arithmetic mean of the surface and the root mean square height (SQ) represents the standard deviation in height. These parameters, derived from the analysis of the AFM images, are representative of the surface roughness of the sample. Results obtained for 1 day of stirring show surface roughness much higher with heights up to 7 μm. For the rest of the cases, the values of SA and SQ are also very high, with slightly lower values for 7 days of agitation. All the samples studied differ greatly from the flatness from the sol–gel recipe where only GLY^27^ is added. The interaction with the CTAB as a function of time is evident.^25^

**Fig. 5:**
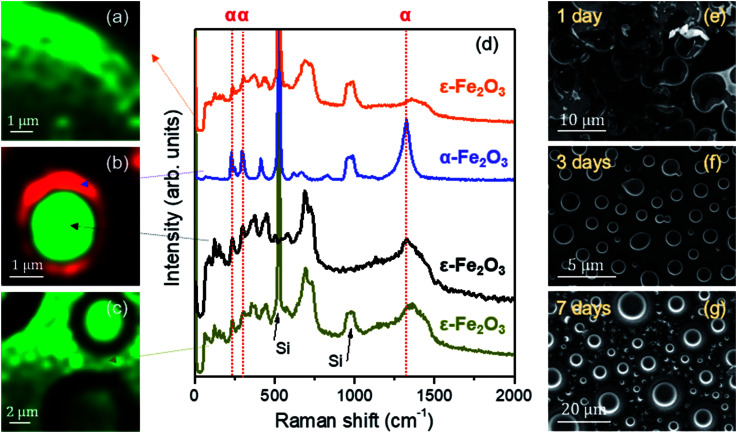
(a–c) Raman intensity images acquired in the *XY* plane on the region presented in Fig. 4 (a–c); average Raman spectra of the iron oxides observed in (a–c). Arrows indicate the areas where the averages are performed. Dashed red lines are added indicating the positions of the vibrational modes associated with the α-Fe_2_O_3_ phase for clarity; (d) average Raman spectra; (e–g) SEM images corresponding to the samples with stirring times of 1, 3 and 7 days.

A compositional characterization of the samples is performed by CRM. The analysis was carried out at the same areas studied by AFM or close to them (Fig. 4), marked with yellow boxes on the optical microscopy images (left panel of Fig. 4). CRM reveals the presence of the ε-Fe_2_O_3_ phase in the three series examined (see Raman spectra in Fig. 5d). The epsilon polymorph is the dominant phase in all the areas studied in the cases of 1 and 7 days (Fig. 4d and f). However, in the 3 days sample, it is concentrated inside the larger islands (Fig. 4e) while the particles that form part of the rest of the coating are pure hematite.^25^ To obtain the Raman intensity images in the *XY* plane, the region of the Raman spectra ranging from 660 to 750 cm^−1^ is integrated and the ε-Fe_2_O_3_ phase is coloured in green. In the case of α-Fe_2_O_3_ phase, the integrated spectral zone ranges from 1310 cm^−1^ to 1340 cm^−1^ and is coloured in red.

From each Raman mapping an averaged Raman spectrum is presented in Fig. 5d. It is worth remembering that the Raman spectra obtained for the three samples are associated with particle sizes confined to the nanoscale. From these spectra it can be deduced that the α-Fe_2_O_3_ phase also appears as a residual manner in the 1 day and 7 days samples. The α-Fe_2_O_3_ bands located at 220, 290 and 1320 cm^−1^ appear superimposed on the Raman spectrum of the ε-Fe_2_O_3_ phase (Fig. 5d). Relative intensities of these hematite Raman bands are similar to the Raman spectra acquired of the samples with compositional percentages of hematite below ∼10%.^26^ Therefore, we obtain ∼90% purity of the epsilon phase in the areas studied by CRM for the 1 day and 7 days samples.

The position of the Raman bands associated with α-Fe_2_O_3_ is indicated by dashed red lines. The lines corresponding to the Raman modes located at 220 and 290 cm^−1^ coincide in the Raman spectra of the 1 day and 7 days samples. On the other hand, as we present in Fig. 4e, the hematite phase in the 3 days sample is clearly identified by the characteristic Raman modes of this iron oxide.^26,34^

Although these Raman spectra have similarities with the spectra obtained for the glycerol-only samples reported in a previous work,^27^ an accurate phase percentage of the different iron oxide polymorphs cannot be assigned. This could be obtained by comparing the relationship of intensities between some vibrational modes attributed to the ε-Fe_2_O_3_ phase and another associated with the α-Fe_2_O_3_ phase, with respect to a calculated purity percentage, for example, with Mössbauer spectroscopy.^35^ This approximation would be valuable if both phases were mixed with the same morphology in the three samples. But, unfortunately, the morphological features of samples vary with the stirring time (Fig. 4 and 5). Therefore, it is not possible to calculate the compositional percentage of the different iron oxide polymorphs. Nevertheless, the purity percentage present in each sample is further estimated by analysing the Mössbauer measurements.

One possible hypothesis for the formation of different polymorphs could lie in the crystal domain size difference. To obtain these values, XRD measurements are performed in grazing incidence mode to achieve a higher surface sensitivity. Different grazing incidence angles (*ω*) are tested and the patterns where a higher signal-to-noise ratio are represented in the ESI.† Diffraction peaks are very broad in the different patterns, indicating that the crystal domains are of few nanometres in size, as already revealed by the Raman measurements. An intense and narrow maximum is detected in all the cases studied, followed by a bulge centred on 55°. This contribution, which is shaded with a grey colour, is associated with the signal of the Si(100) substrate in the grazing incidence mode, whose diffraction peak positions located between 50 and 52° vary when *ω* is modified. For each studied *ω* different reflections of the substrate are promoted. The XRD patterns for 1 and 7 days of agitation indicate that the only phase present in these samples is ε-Fe_2_O_3_ according to the resolution of the measurement conditions.^36^ However, for the 3 days sample, α-Fe_2_O_3_ phase^37^ and traces of reflections from the ε-Fe_2_O_3_ phase can be distinguished. Only the reflections corresponding to the (131) and (123) planes can be attributed uniquely to ε-Fe_2_O_3_. On the other hand, the highest diffraction peak for ε-Fe_2_O_3_ and hematite is 33.05° and 33.16° respectively.^36,37^ This slight angular displacement is detected in the 3 days sample pattern. The crystal domain sizes are calculated using the Scherrer's relation with a form factor of 0.94 assuming that the particles are spherically shaped. The value obtained for the cases of 1, 3 and 7 days for the ε-Fe_2_O_3_ phase are respectively ∼10.9(5), ∼12.6(9) and ∼13.2(3) nm. This means that the particles/islands observed in the 3 and 7 days samples are probably agglomerates formed by sub-grains. With respect to the value calculated for the α-Fe_2_O_3_ crystal domain size of the 3 days sample, is ∼12.5(3) nm. This result is similar to the one obtained for the ε-Fe_2_O_3_ phase.

**Fig. 6:**
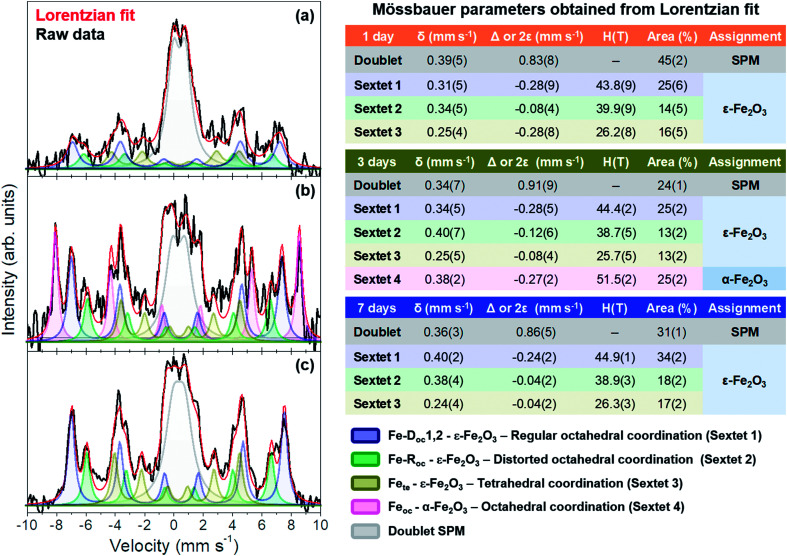
Mössbauer spectra collected in reflection mode at 300 K for samples with (a) 1, (b) 3 and (c) 7 days of magnetic stirring time. Obtained Mössbauer parameters are presented to the right of the (a–c). The legend at the right bottom refers to each sextet and doublet corresponding to the ε- and α-Fe_2_O_3_ phases.

Fig. 6 shows the integral conversion electron Mössbauer (ICEMS) spectra recorded at room temperature from the three samples. Very long acquisition times were needed to record the spectra with reasonable statistics. It was observed (Fig. 6) that the signal-to-noise ratio increased as the stirring time used to prepare the samples increased. This is surely related with a larger concentration of iron present in the deposited films as the stirring time increases and correlates well with the AFM observations that showed also an increase in the number and height of islands with the agitation time. The spectra contain various magnetic contributions together with a relative intense paramagnetic doublet. Fig. 6 collects the Mössbauer parameters obtained from the fit of the spectra. All the samples contain the three sextets characteristic of the ε-Fe_2_O_3_ phase.^27,38^ Additionally, the sample prepared after 3 days of magnetic stirring contained a α-Fe_2_O_3_ contribution.^39^ The parameters of the paramagnetic doublet are typical of Fe^3+^ in octahedral oxygen coordination^40,41^ and might correspond to a fraction of superparamagnetic ε-Fe_2_O_3_/α-Fe_2_O_3_ particles.

Inspection of Fig. 6 show that the three samples are rich in ε-Fe_2_O_3_ and that the superparamagnetic contribution decreases substantially from 45% (1 day sample) to 31% (7 days sample) (Fig. 6). Therefore, the samples with a higher amount of pure ε-Fe_2_O_3_ are obtained with a stirring time of 7 days and, as stated above, this has to be related with the larger particle sizes obtained after 7 days as compared with those obtained after 1 and 3 days of stirring time. In contrast, the presence of hematite is only detected in the 3 days sample, a fact that has been also corroborated by XRD (ESI†). We would like to point out, however, that this phase has been detected, in very small amounts, by CRM in all the other samples (Fig. 5a–d). This would suggest that the films could contain a fraction of small particle hematite which would contribute to the observed Fe^3+^ paramagnetic doublet and that would go undetected by XRD.

**Fig. 7:**
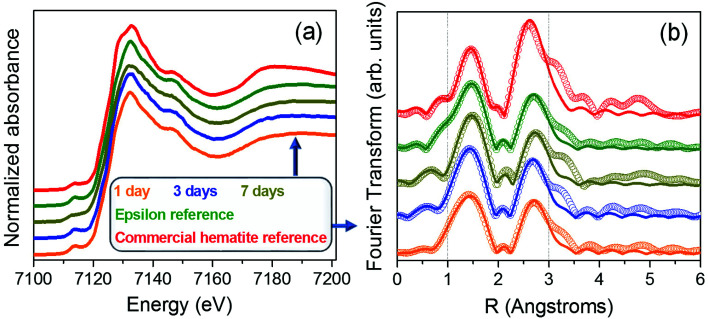
(a) Normalized Fe K-edge XANES spectra disposed in cascade of samples with 1 day (orange), 3 days (blue) and 7 days (dark yellow) of magnetic stirring time, ε-Fe_2_O_3_ reference sample (deep green) and α-F_2_O_3_ reference (red). The second resonance of the X-ray scattering is asterisked; (b) Fourier transform magnitude (open circles) of Fe K-edge EXAFS (*k*^2^-weighted) and fits carried out in the range comprised between 1 and 3 Å (continuous lines); colour code the same of (a). Vertical discontinuous lines indicate the range of the fit in the *R*-space.

To complete this work, a compositional analysis of different iron oxide phases present in the samples was also performed by XAS. Samples grown in this work show great similarities with the reference rich in ε-Fe_2_O_3_ phase.^27^Fig. 7a shows the X-ray absorption near edge structure (XANES) measurements recorded from the samples prepared at different stirring times as well as from a commercial hematite reference and a high purity (∼100%) ε-Fe_2_O_3_ sample. No variations in the absorption edge position are detected, indicating that the samples are formed by oxide phases in which iron is in the oxidation state 3+.^42,43^ No major differences are detected in the XANES zone between the 1, 3 and 7 days of agitation time compared to the ε-Fe_2_O_3_ reference sample, suggesting that they have similar compositional percentages.

The local environment of the iron atoms was analysed for each sample by extended X-ray absorption fine structure (EXAFS). The Fourier transform of the Fe K-edge EXAFS recorded from all the samples are shown in Fig. 7b. The EXAFS signal *χ*(*k*) was weighted by *k*^2^ and fitted in *R*-space in the range 1 to 3.0 Å using the FEFFIT code.^44,45^ For the model, a single scattering is considered and two shells have been evaluated. The first one is composed by Fe–O interatomic distances while for the second shell only Fe–Fe interatomic distances are taken into account. The Debye–Waller factor and energy shift obtained from the fit of the ε-Fe_2_O_3_ reference have been fixed for the rest of the samples (Table 2i). Regarding the α-Fe_2_O_3_ phase, the obtained fit parameters correspond to those reported in other works.^46,47^ On the other hand, the EXAFS spectra fit well for the rest of the samples with errors tending to be minimized as the agitation time increases up to 7 days (Table 2ii). Considering the similar values obtained for the samples compared with those of the ε-Fe_2_O_3_ reference, we can assure that samples present a high degree of purity in ε-Fe_2_O_3_ polymorph independently of stirring time.

(i) Formal coordination (*N*), interatomic Fe–O and Fe–Fe bond lengths (*R*) and Debye–Waller factors (*σ*^2^) obtained by EXAFS analysis with uncertainties expressed in brackets for α- and ε-Fe_2_O_3_ references.^27,47^ (ii) EXAFS parameters calculated for the different samples prepared varying the stirring time

**Table:** 

(i) Fit reference sample	ε-Fe_2_O_3_		α-Fe_2_O_3_	
Shell	Fe–O	Fe–Fe	Fe–O	Fe–Fe
*N*	6.2(5)	4.1(3)	6.0(1)	4.1(2)
*R*	1.928(5)	3.083(7)	1.971(2)	3.022(9)
*σ* ^2^	0.0109(2)	0.0114(3)	0.0111(8)	0.0076(4)

**Table:** 

(ii) Sample	1 day		3 days		7 days	
Shell	Fe–O	Fe–Fe	Fe–O	Fe–Fe	Fe–O	Fe–Fe
*N*	5.5(5)	3.8(4)	6.2(5)	4.1(3)	6.1(3)	3.8(3)
*R*	1.937(5)	3.098(6)	1.928(5)	3.083(7)	1.927(9)	3.108(2)

## Conclusions

This study shows the importance of controlling the agitation time during the synthesis of ε-Fe_2_O_3_ nanoparticles produced by sol–gel method. In this research we have studied morphologically, structurally and compositionally samples containing a high percentage of pure ε-Fe_2_O_3_ (>75%) with different microstructures as a coating on Si(100) substrates. This is the first time that different micropatterns are obtained simply varying the agitation time of the sol–gel recipe thanks to the simultaneous addition of GLY and CTAB. The densified coatings show different morphologies and phase compositions depending on their magnetic stirring time. The average crystalline domain sizes for the ε-Fe_2_O_3_ phase are in the 10 nm range for all cases. The Mössbauer analysis indicates that the three samples are rich in ε-Fe_2_O_3_. The short range crystalline order and the immediate environment of the iron sites are studied by means of XAS experiments at room temperature using the fluorescence detection mode.

Hematite appears in the three sample series but the influence in the morphology and the microstructure favours larger island sizes for 3 days samples. The highest purity and the highest crystallinity for the ε-Fe_2_O_3_ polymorph is obtained for 7 days of stirring time.

This investigation manifests the drastic influence that one polyalcohol and one surfactant have in the sol–gel synthesis of the samples. This effect can open a field of investigation where the use of other steric agents could change the shape, the morphology and/or the roughness of the epsilon iron oxide samples depending on the particular application.

## Conflicts of interest

The authors declare that there is no conflict of interest regarding the publication of this article.

## Supplementary Material

RA-009-C9RA03283E-s001
