# Supplementary material for: Self-assembly of iron oxide precursor micelles driven by magnetic stirring time in sol–gel coatings
Source: RSC Adv. 2019 Jun 4;9(31):17571–80. doi: 10.1039/c9ra03283e (PMC9064545; doi:10.1039/c9ra03283e)
Supplement: RA-009-C9RA03283E-s001 [file RA-009-C9RA03283E-s001.pdf]

Electronic Supplementary Material (ESI) for RSC Advances.
This journal is © The Royal Society of Chemistry 2019

Supporting information

Self-assembly of iron oxide precursor micelles driven by magnetic stirring time in sol-gel coatings

J. López-Sánchez[1*], A. Serrano[2,3,4], A. del Campo[2], M. Abuín[5], E. Salas-Colera[3,4], A. Muñoz-Noval[3,4,7], G. R. Castro[3,4], J. de la Figuera[6], J. F. Marco[6], P. Marín[1,7], N. Carmona[1,7], O. Rodríguez de la Fuente[1,7]

[1] Instituto de Magnetismo Aplicado, UCM-CSIC-ADIF, 28230 Madrid, Spain
[2] Instituto de Cerámica y Vidrio, ICV-CSIC, 28049 Madrid, Spain
[3] SpLine, Spanish CRG BM25 Beamline, ESRF-The European Synchrotron, 38000 Grenoble, France
[4] Instituto de Ciencia de Materiales de Madrid, ICMM-CSIC, 28049 Madrid, Spain
[5] Instituto de Sistemas Optoelectrónicos y Microtecnología, ISOM-UPM, 28040 Madrid, Spain
[6] Instituto de Química Física "Rocasolano", IQFR-CSIC, 28006 Madrid, Spain
[7] Departamento de Física de Materiales, Universidad Complutense de Madrid (UCM), 28040 Madrid, Spain

*Corresponding author: jesus.lopez@ucm.es

Morphological and structural properties:

AFM measurements

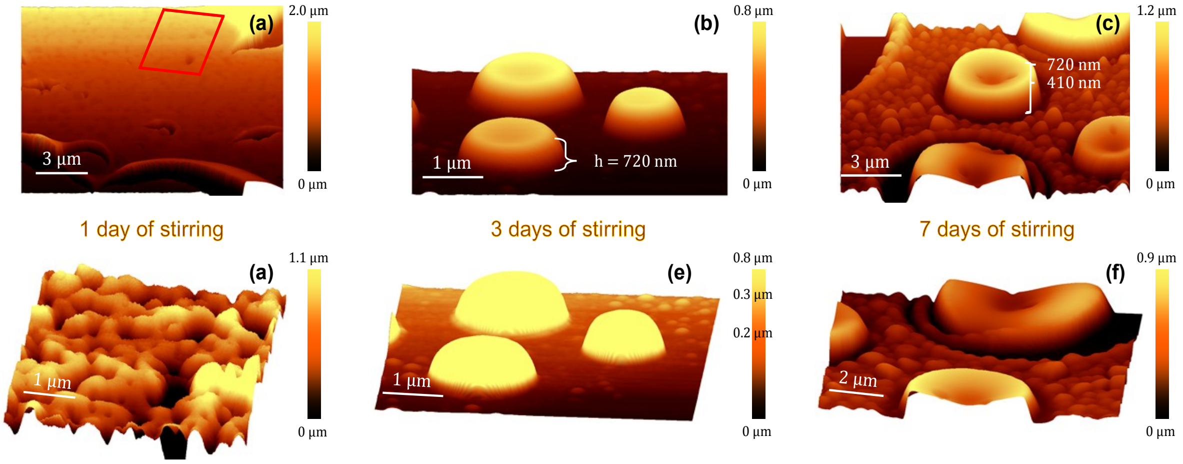

Figure S1. AFM images obtained with the CRM system presented with perspective in height of the surface and islands formed for samples prepared at 960 ºC for (a) 1 day, (b) 3 days and (c) 7 days of magnetic stirring, respectively. Figure (d) represents the swept area indicated with a red rectangle in the upper image, Figures (e-f) represent another degree of inclination, and another contrast compared to Figures (b-c) respectively.

XRD results

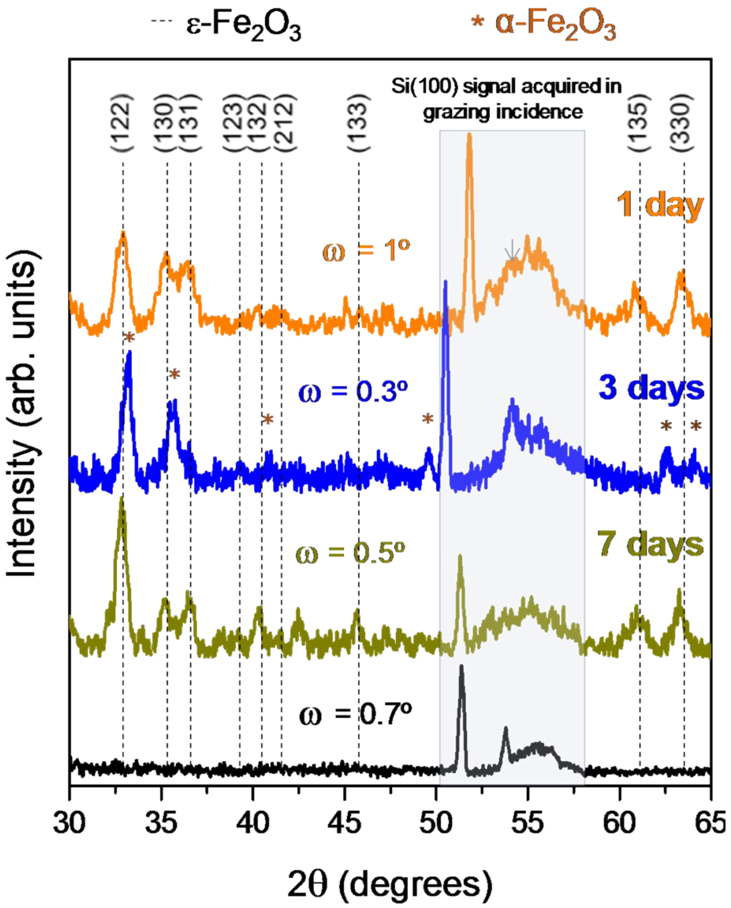

Figure S2. XRD patterns of samples related to 1, 3 and 7 days of magnetic agitation, respectively. The X-ray diffraction peaks characteristic of the ε-Fe_2O_3 phase are indexed and indicated with black dotted lines. The peak diffractions attributed to the hematite phase are indicated with red asterisks. The grey shaded area corresponds to the signal contribution from the Si(100) substrate. ω represent the X-ray incidence grazing angle.
